# Comparison of hepatitis B vaccine efficacy in Japanese students: a retrospective study

**DOI:** 10.1186/s12199-019-0837-1

**Published:** 2019-12-26

**Authors:** Masanori Ogawa, Dai Akine, Teppei Sasahara

**Affiliations:** 1 Health Service Center, Jichi Medical University, 3311-1, Yakushiji, Shimotsuke, 329-0498 Japan; 2 Division of Clinical Infectious Diseases, Department of Infection and Immunity, Jichi Medical University, 3311-1, Yakushiji, Shimotsuke, 329-0498 Japan

**Keywords:** Bimmugen®, Hepatitis B vaccine, Heptavax-II®, Intramuscular, Subcutaneous

## Abstract

**Background:**

Two types of recombinant hepatitis B virus (HBV) vaccines are available in Japan. One type uses the antigen from genotype A (Heptavax-II®) and the other uses the antigen from genotype C (Bimmugen®). Potential differences in productivity of the hepatitis B virus surface (HBs) antibody between vaccines have not been studied in detail. We investigated the acquired level of immunity against HBV in association with two vaccines, their administration routes, and patient sex. We present the appropriate inoculation method based on the characteristics of each vaccine.

**Methods:**

Data of 1135 medical and nursing students (481 men and 651 women) were used, each of whom was unvaccinated prior to recruitment and subsequently vaccinated three times prior to the study. The vaccine type and administration route differed according to the university department and enrolling year. The students were categorized into the following three groups: Bimmugen®-subcutaneous group, Heptavax-II®-subcutaneous group, and Heptavax-II®-intramuscular group. The total and sex-segregated positive rates of the HBs antibody among the three groups were compared using Pearson’s chi-square test. The effect of time between the HBs antibody test and vaccine administration on the HBs antibody level was also analyzed similarly.

**Results:**

The Bimmugen®-subcutaneous group showed the highest positive HBs antibody rate (92.0%) among the three groups. In the Heptavax-II® group, the positive rate was 66.3% in the subcutaneous injection group and 89.1% in the intramuscular injection group. There was a significant difference among these three groups. In terms of sex, women showed a significantly higher average positive rate than men in each group. In terms of effect of time between the HBs antibody test and vaccine administration, no significant differences were observed.

**Conclusions:**

Bimmugen® is associated with more effective HBs antibody production than Heptavax-II® in Japanese students. However, the Heptavax-II® vaccine is an appropriate choice for HBV vaccination in areas where HB is caused predominantly by HBV genotype C. With both vaccines, women tended to acquire more immunogenicity than men. Intramuscular injection may be the preferred administration route due to the possibility of local reactions.

## Background

Hepatitis B vaccine against the hepatitis B virus (HBV), a blood-borne pathogen, is recommended for all infants and children up to the age of 18 years by the World Health Organization and the US Centers for Disease Control and Prevention. Japan also adopted the hepatitis B vaccine for infants less than 1 year old as a universally required vaccine in 2016 [1]. In the future, most Japanese citizens will be immunized against HBV. However, at present, health care workers (HCWs) in Japan have not been vaccinated against HBV as universal vaccination. As HCWs are at risk of exposure to HBV, an HBV vaccine is recommended for them [2–5]. Medical and nursing students practice at hospitals and are considered to be equal to HCWs from an infection-control standpoint. Therefore, new medical and nursing school students should receive an HBV vaccine.

Two types of recombinant HBV vaccines are available in Japan. One type uses the antigen from genotype A (Heptavax-II®, Merck, Kenilworth, NJ, USA) and the other uses the antigen from genotype C (Bimmugen®, KM Biologics, Kumamoto, Japan). Both can be administered intramuscularly or subcutaneously, and there is no generalization of the administration route. These vaccines are considered to have the same clinical effect. Although the intramuscular route of administration was not studied for Bimmugen® and Heptavax-II® and the exclusion of the effect of past vaccination was unclear, the acquired immunity levels after the two vaccinations were different, which were also dependent on sex [6]. Moreover, HB caused by HBV genotype C was recently reported to be predominant in Asia, including Japan [7, 8]. Therefore, it is important to investigate the acquired immunity level associated with HBV vaccines according to vaccine type, administration route, and sex.

In this study, we retrospectively investigated the acquired immunity level associated with HBV vaccines for medical and nursing students according to vaccine type, administration route, and student sex, excluding the effect of past vaccination. From these results, we present the appropriate inoculation method based on the vaccine and patient characteristics.

## Methods

### Participants, vaccination schedule, and vaccine

Data of 1135 medical and nursing students (481 men and 651 women) were used retrospectively. All students were enrolled in Jichi Medical University from 2013 to 2017 (medical students) and 2015 to 2017 (nursing students). The age distribution was 19–30 years (median 20 years), and all students were Japanese. None reported illness related to immunogenicity.

During the first school year at the university, most students receive an HBV vaccine to prevent HB infection. Students who had already received an HBV vaccine before entry to the university were exempted from this vaccination and this study, and so were students who had vaccine allergy.

According to the guideline [9, 10], the HB vaccine is administered as a three-dose series with a 0-, 1-, and 6-month schedule. Nursing students have their first vaccination in May, and medical students have their first vaccination in September. The levels of the HBs antibody were measured in April of the subsequent year according to the Clinical Laboratory Improvement Amendments (CLIA) method in the laboratory of LSI Medience (Tokyo, Japan). Although the HBs antibody titer of each student was determined, we classified the students into positive (+) and negative (−) groups based on a report, according to which an HBs antibody titer of above 10 mIU/mL was considered a protective antibody titer [11].

Bimmugen® (genotype C-derived vaccine) and Heptavax-II® (genotype A-derived vaccine) are available in Japan. In this university, Bimmugen® (0.5 mL) has been used exclusively and was administered subcutaneously even before the study. This route was chosen because many students can be vaccinated within a short timeframe, and subcutaneous injection can be done more quickly than intramuscular injection. When the students expose the point for intramuscular injection (deltoid muscle), they have to roll their sleeve up to the shoulder, which slows down the process, especially in winter. Although it has been reported that immunogenicity is greater with intramuscular than with subcutaneous injection [12], in the present study, over 90% of students who were subcutaneously vaccinated with Bimmugen® had immunity against HBV even before this study.

However, Bimmugen® was not in stable supply due to a manufacturing issue in 2015; therefore, subcutaneous injection of Heptavax-II® (0.5 mL) was adopted in the latter part of 2015. In 2015, subcutaneous injections of Bimmugen® for nursing students and subcutaneous injection of Heptavax-II® for medical students were carried out.

After vaccination, the HBs antibody positive rate after Heptavax-II® was considerably lower than that after Bimmugen® (Bimmugen®-subcutaneous group 92.0%, Heptavax-II®-subcutaneous group 66.3%) (Table 2). Furthermore, Bimmugen® supply continued to be unstable even in 2017, as an earthquake in April 2016 in Kumamoto, Japan, damaged the manufacturing facility. In addition, there is evidence that the intramuscular injection muscular route is associated with better immune response than the subcutaneous injection route [12]. Therefore, in 2017, Jichi Medical University switched from subcutaneous injection of Heptavax-II® to intramuscular injection of Heptavax-II® although the administration of the vaccine via this route is more time consuming.

The vaccines and administration routes used according to the admission year are summarized in Table 1. All students were categorized into the following three groups: Bimmugen®-subcutaneous group, Heptavax-II®-subcutaneous group, and Heptavax-II®-intramuscular group.

**Table 1:** Vaccines and the administration route according to the admission year

Admission year	Department	Vaccine	Administration route
2017	Medical	Heptavax-II®	Intramuscular
	Nursing	Heptavax-II®	Intramuscular
2016	Medical	Heptavax-II®	Subcutaneous
	Nursing	Heptavax-II®	Subcutaneous
2015	Medical	Heptavax-II®	Subcutaneous
	Nursing	Bimmugen®	Subcutaneous
2014	Medical	Bimmugen®	Subcutaneous
2013	Medical	Bimmugen®	Subcutaneous

### Time between HBs antibody test and vaccine administration

As a rule, the HBs antibody level should be tested 1–2 months after the final vaccination [10, 13]. In this study, the HBs antibody was measured in April, the second school year for all students. Therefore, the HBs antibody test was performed 2 months after the last vaccination in medical students and 5 months after the last vaccination in nursing students.

### Statistical analyses and ethics

We used Mann–Whitney *U* test to compare age distribution among the three groups. Comparison among each group by sex was carried out similarly.

To estimate the level of HBs antibody productivity according to vaccine type and administration route, the positive rate of the HBs antibody and the sex-segregated positive rate of the HBs antibody among the three groups were compared using Pearson’s chi-square test. Comparison of the HBs antibody productivity between men and women in each group was performed similarly. The positive rate of the HBs antibody according to the time between vaccination and the HBs antibody test was also analyzed using Pearson’s chi-square test.

The software program JMP 10 (SAS Institute, Cary, NC, USA) was used for these analyses. Values of *p* < 0.05 were regarded as statistically significant.

Regarding ethics, we gave the target students the opportunity to opt out of the study before it began. This study was approved by the Ethics Committee of Jichi Medical University (approval no. 18-033).

## Results

The number of subjects in the Bimmugen®-subcutaneous group, the Heptavax-II®-subcutaneous group, and the Heptavax-II®-intramuscular group was 514, 373, and 247, respectively. The age range in the Bimmugen®-subcutaneous, Heptavax-II®-subcutaneous, and Heptavax-II®-intramuscular group was 19–25 (median 20 years), 19–30 (median 20 years), and 19–27 years (median 20 years), respectively. There were no significant differences among the groups. Moreover, there were no significant differences in age distribution between males and females (Table 2).

**Table 2:** Positive rate of HBs antibody after HB vaccination

Vaccine Administration route	Total number	Age range, median (IQR)	HBs antibody			Positive rate (%)	Sex	Total number	Age range, median (IQR)	HBs antibody		Positive rate (%)
			Median (IQR)	(+)	(−)					(+)	(–)	
Bimmugen® subcutaneous	514	19-25, 20 (19–21)	84.9 (34.5–217) mIU/mL	473	41	92.^*^	Men	204	19–25, 20 (19–21)	183	21	89.^†^
							Women	310	19–22, 19 (19–19)	290	20	93.^‡^
Heptavax-II® subcutaneous	373	19–30, 20 (19–21)	28.7 (5–216) mIU/mL	248	125	66.3^*^	Men	180	19–30, 20 (19–21)	102	72	56.^†^
							Women	193	19–22, 19 (19–19)	146	7	75.^‡^
Heptavax-II® intramuscular	247	19–27, 20 (19–21)	190, (41.6–534) mIU/mL	220	27	89.^*^	Men	97	19–27, 20 (19–21)	79	18	81.4^†^
							Women	150	19–22, 19 (19–20)	141	9	94.^‡^

*There was a significant difference among the Bimmugen® subcutaneous, Heptavax-II® subcutaneous, and Heptavax-II® intramuscular groups (*p* < 0.05)

^†,‡^There were significant differences among the Bimmugen® subcutaneous, Heptavax-II® subcutaneous, and Heptavax-II® intramuscular groups in both men and women (*p* < 0.05)

The median HBs antibody titers in each group were 84.9 mIU/mL (IQR 34.5–217 mIU/mL) in the Bimmugen®-subcutaneous group, 28.7 mIU/mL (IQR 5–216 mIU/mL) in the Heptavax-II®-subcutaneous group, and 190 mIU/mL (IQR 41.6–534 mIU/mL) in the Heptavax-II®-intramuscular group. An antibody titer above 10 mIU/mL was considered to be positive [10]; the positive rate of the HBs antibody obtained after HB vaccination is summarized in Table 2.

The Bimmugen®-subcutaneous group showed the highest positive rate (92.0%) among the three groups (the Bimmugen®-subcutaneous, Heptavax-II®-subcutaneous, and Heptavax-II®-intramuscular groups), despite being administered via subcutaneous injection. For the Heptavax-II® type vaccine, the positive rate in the subcutaneous injection group was 66.3% and that in the intramuscular injection group was 89.1%. There were significant differences among those three groups.

The sex-segregated positive rate of the HBs antibody among the three groups showed significant differences in both men and women. Moreover, women showed a significantly higher positive rate than men in each group.

The positive rate of the HBs antibody according to the time between vaccination and the HBs antibody test is summarized in Table 3. As mentioned above, the HBs antibody productivity differed according to the vaccine type, administration route, and sex. Therefore, the positive rate of the HBs antibody according to the time between vaccination and the HBs antibody test was analyzed by vaccine, administration route, and sex. The number of men who received the HBs antibody test 5 months after each vaccination was small (less than 10); therefore, the influence of time on the HBs antibody was analyzed using the women’s data. Regarding the positive rate of the HBs antibody, there were no significant differences between 2 and 5 months after vaccination in all the groups.

**Table 3:** Hepatitis B virus surface (HBs) antibody positive rate according to the time between vaccination and the HBs antibody test

Vaccine administration route	HBs antibody 2 or 5 months after vaccination											
	2 months						5 months					
	Total		Men		Women		Total		Men		Women	
	(+)	(−)	(+)	(−)	(+)	(−)	(+)	(−)	(+)	(−)	(+)	(−)
Bimmugen® subcutaneous	266	27	175	19	91	8	207	14	8	2	199	12
Positive rate	90.8%		90.2%		91.9%^*^		93.7%		80.0%		94.3%^*^	
Heptavax-II® subcutaneous	161	100	99	76	62	24	87	25	3	2	84	23
Positive rate	61.7%		56.6%		72.1%^†^		77.0%		60.0%		78.5%^†^	
Heptavax-II® intramuscular	104	19	76	17	28	2	116	8	3	1	113	7
Positive rate	84.6%		81.7%		93.3% ^‡^		93.5%		75.0%		94.2%^‡^	

^*,†,‡^There were no significant differences between 2 and 5 months after vaccination in all the groups (*p* > 0.05)

## Discussion

There are reports that the productivity of the HBs antibody by HB vaccination is higher with intramuscular administration than with subcutaneous administration [12]. Our results corroborated these findings, as we observed this tendency with the Heptavax-II® vaccine in both HBs antibody titer and positive rate. The HBs antibody titer in the Heptavax-II®-intramuscular group was considerably higher than that in the Bimmugen®-subcutaneous group. However, the positive rate in the Bimmugen®-subcutaneous group was higher than that in the Heptavax-II®-intramuscular group or almost at the same level. Our study showed that Bimmugen® produces protective HBs antibody (> 10 mIU/mL) effectively than Heptavax-II® in this population of Japanese students.

The HBs antibody productivity was influenced by sex, as the HBs antibody positive rate in each group was significantly lower in men than in women. Therefore, our findings suggest that, especially for Japanese male students, Bimmugen® vaccination should be recommended.

In the present study, we did not investigate the mechanisms by which HBs antibody productivity differed between vaccine stains and sex. However, there is a report that single nucleotide polymorphism in HLA (*HLA-DR-DQ* and *BTNL2* in HLA class II and III regions) is associated with response to HB vaccination [14]; therefore, the differences in HBs antibody productivity from vaccine strains and sex may be due to genetic polymorphism. The difference between sexes might be due to gonadal hormones playing a role in immunogenicity [15].

Bimmugen® and Heptavax-II® are both recombinant HBV vaccines. Heptavax-II® is a genotype A-derived vaccine, and Bimmugen® is a genotype C-derived vaccine. There are reports that vaccines with genotype A- or C-derived HBs antigens have the ability to induce cross-genotype immunity against HBV infection [16, 17]. It has also been reported that a high HBs antibody titer is required to prevent HBV infection in non-vaccine genotypes [16].

Epidemiological studies indicate that HB caused by HBV genotype C is predominant in Asia, including Japan [7, 8]. However, acute HB caused by HBV genotype A has been increasing in Japanese young adults in urban areas because it is imported from foreign countries and spread as a sexually transmitted disease [17–19]. The National Institute of Infectious Disease in Japan has attributed 70% of acute hepatitis B infection to sexual contact [20]. There are reports that HB caused by HBV genotype A develops into chronic hepatitis more often than HB caused by HBV genotype C [21, 22]. As mentioned above, although genotype A- or C-derived HBs antigens have the ability to induce cross-genotype immunity against HBV infection, a high HBs antibody titer may be needed to prevent HBV infection in non-vaccine genotypes [16, 17]. Therefore, Heptavax-II® is an appropriate choice depending on data such as the geographic area and characteristics of the subjects requiring vaccination in order to match the vaccine and suspected genotypes.

It is recommended that the HBs antibody test be performed 1–2 months after the final vaccination [10, 13]. In our study, the HBs antibody positive rate was determined 2 and 5 months after vaccination. As the HBs antibody titer decreases with time [23], there was a concern that the positive rate difference between each group may be influenced by the time between the HBs antibody test and vaccine administration. To estimate the influence of time, we compared the positive rate between 2 and 5 months after vaccination in each group using data from women; data from men were excluded due to a small sample size. There were no significant differences in the positive rate between 2 and 5 months after vaccination among the groups. These results suggest that the HBs antibody positive rate was not influenced by the time between the HBs antibody test and vaccine administration. Therefore, the differences in the HBs antibody positive rate were attributed to the vaccine strains; Bimmugen® produces protective HBs antibodies more effectively than Heptavax-II® in Japanese students.

As a vaccination administration route, intradermal injection may be considered in addition to the intramuscular and subcutaneous routes. Some studies have reported that the administration of HBV recombinant vaccine by the intradermal route is more effective than the intramuscular route [24]. However, as the intradermal injection of HBV vaccine is not permitted officially in Japan, the intradermal injection was not performed in our university.

HBV vaccines have been demonstrated to be safe [25]. However, some studies have reported that aluminum-adjuvanted vaccines should be administered by intramuscular injection because of local reactions to the adjuvant [12, 26]. Bimmugen® and Heptavax-II® are both aluminum-adjuvanted vaccines. In this study, the Bimmugen®-subcutaneous group showed almost over 90% of HBs antibody positive rate; however, intramuscular injection may be considered due to the possibility of local reactions. On the contrary, Heptavax-II® showed a significant difference in HBs antibody positive rate between the subcutaneous and intramuscular groups. Therefore, intramuscular injection should be the preferred route of administration of Heptavax-II® based on positive immunity.

There were some limitations in this study. First, the subjects were all young adults; therefore, the results may not be the same in other generational populations. Second, we did not consider Bimmugen® intramuscular injection in this study. However, with Bimmugen®, the productivity of the HBs antibody seems to be greater with intramuscular injection than with subcutaneous injection.

## Conclusions

Bimmugen® produces protective HBs antibody more effectively than Heptavax-II® in Japanese students. However, the Heptavax-II® vaccine is also an appropriate choice for HBV vaccination for some geographic area and subjects. Intramuscular injection may be the preferred administration route due to the possibility of local reaction. With both vaccines, women tended to acquire more immunogenicity than men.

## Data Availability

The datasets used and/or analyzed in this study are available from the corresponding author by reasonable request.

## Abbreviations

CLIA: Clinical Laboratory Improvement Amendments
HB: Hepatitis B
HBs: Hepatitis B virus surface
HBV: Hepatitis B virus
HCW: Health care worker
