# Correction to: Comparison of hepatitis B vaccine efficacy in Japanese students: a retrospective study

**DOI:** 10.1186/s12199-020-0843-3

**Published:** 2020-02-03

**Authors:** Masanori Ogawa, Dai Akine, Teppei Sasahara

**Affiliations:** 1 Health Service Center, Jichi Medical University, 3311-1, Yakushiji, Shimotsuke, 329-0498 Japan; 2 Division of Clinical Infectious Diseases, Department of Infection and Immunity, Jichi Medical University, 3311-1, Yakushiji, Shimotsuke, 329-0498 Japan

**Correction to: Environ Health Prev Med (2019) 24:80**

**https://doi.org/10.1186/s12199-019-0837-1**

Following publication of the original article [1], the authors spotted an error in their paper concerning the positive rate in the right side in Table 2.

That is, 89.7 not 89., 93.5 not 93., 56.7 not 56., 75.3 not 75. and 94.0 not 94..

The original article has been corrected. The correct presentation of Table 2 is shown below.

**Table 2:** Positive rate of HBs antibody after HB vaccination

Vaccine Administration route	Total number	Age range, median (IQR)	HBs antibody			Positive rate (%)	Sex	Total number	Age range, median (IQR)	HBs antibody		Positive rate (%)
			Median (IQR)	(+)	(−)					(+)	(−)	
Bimmugen® subcutaneous	514	19–25, 20 (19–21)	84.9 (34.5–217) mIU/mL	473	41	92.0 ^*^	Men	204	19–25, 20 (19–21)	183	21	89.7 ^†^
							Women	310	19–22, 19 (19–19)	290	20	93.5 ^‡^
Heptavax-II® subcutaneous	373	19–30, 20 (19–21)	28.7 (5–216) mIU/mL	248	125	66.3 ^*^	Men	180	19–30, 20 (19–21)	102	72	56.7 ^†^
							Women	193	19–22, 19 (19–19)	146	47	75.3 ^‡^
Heptavax-II® intramuscular	247	19–27, 20 (19–21)	190 (41.6–534) mIU/mL	220	27	89.1 ^*^	Men	97	19–27, 20 (19–21)	79	18	81.4 ^†^
							Women	150	19–22, 19 (19–20)	141	9	94.0 ^‡^

^*^ There was a significant difference among the Bimmugen® subcutaneous, Heptavax-II® subcutaneous, and Heptavax-II® intramuscular groups (*p* < 0.05)

^†,‡^ There were significant differences among the Bimmugen® subcutaneous, Heptavax-II® subcutaneous, and Heptavax-II® intramuscular groups in both men and women (*p* < 0.05)
